# Task-Difficulty Homeostasis in Car Following Models: Experimental Validation Using Self-Paced Visual Occlusion

**DOI:** 10.1371/journal.pone.0169704

**Published:** 2017-01-13

**Authors:** Jami Pekkanen, Otto Lappi, Teemu H. Itkonen, Heikki Summala

**Affiliations:** 1 Cognitive Science, University of Helsinki, Helsinki, Finland; 2 Transportation Engineering, Department of Built Environment, Aalto University, Helsinki, Finland; 3 Traffic Research Unit, University of Helsinki, Helsinki, Finland; University of Muenster, GERMANY

## Abstract

Car following (CF) models used in traffic engineering are often criticized for not incorporating “human factors” well known to affect driving. Some recent work has addressed this by augmenting the CF models with the Task-Capability Interface (TCI) model, by dynamically changing driving parameters as function of driver capability. We examined assumptions of these models experimentally using a self-paced visual occlusion paradigm in a simulated car following task. The results show strong, approximately one-to-one, correspondence between occlusion duration and increase in time headway. The correspondence was found between subjects and within subjects, on aggregate and individual sample level. The long time scale aggregate results support TCI-CF models that assume a linear increase in time headway in response to increased distraction. The short time scale individual sample level results suggest that drivers also adapt their visual sampling in response to transient changes in time headway, a mechanism which isn’t incorporated in the current models.

## Introduction

As in all complex natural tasks, appropriate allocation of attention is crucial for successfully driving a vehicle; failure to do so due to a secondary task or drowsiness is estimated to contribute to almost half of all crash and near-crash events [1]. But even if distraction is often involved in the occurrence of a crash, clearly the vast majority of all episodes of momentary distraction on the road do not cause an accident. Although in some cases this may be sheer luck, most of the time the drivers’ ability to adapt their attention and behavior for a given situation ensures successful driving with only partial sensory and cognitive resources. Studies have shown that drivers successfully balance the attentional resources between driving and a secondary tasks using compensatory behavior, e.g. lowering driving speed or pausing conversation in a demanding driving situation [2–4].

Within traffic psychology, the question of how this mechanism works has been subject of extensive discussion for decades (for review of the history see [5] Chapter Two). Much of the debate has revolved around a subjective estimate, or feeling, of *risk*, and how drivers balance their different goals to keep the risk at acceptable levels. However, the risk-based formulations have proven quite problematic to state in quantitative form. An influential model arisen from this discussion is the task-capability interface (TCI) of Fuller [6], which reframes this balance as *task-difficulty homeostasis*: Drivers maintain a preferred level of *task difficulty* which is a difference between *task demand* and *capability*. Importantly for the purposes of quantitative modeling, sidestepping the problematic issue of risk makes the theory more amenable to operationalization.

Although well established in traffic psychology, these mechanisms have traditionally been overlooked by most car following models in traffic engineering, which has raised considerable criticism [7–9] (for a review of human factors in CF models see [10]). As a response to this criticism, the TCI has recently been incorporated in car following models by Hoogendoorn et al. [11] and Saifuzzaman et al. [12]. Both propose that parameters of car following vary as functions of driver capability, so as to maintain the task difficulty at a peferred level. For example, a drop in driver capability due to distraction causes their preferred time headway to the leading vehicle to rise, which lowers the task’s demand and thus maintains a preferred level of task difficulty. However, neither proposal includes an operationalization for capability, and thus lack a direct quantitative validation of the TCI formulation.

Time headway provides quite a natural index for *task demand* in a car following task. It’s often discussed as the main variable drivers control during car following, and has been shown in multiple studies to increase as capability drops [6]. Time headway is also directly measurable and a central measure in traffic engineering.

For driver *capability*, however, such natural operationalization is not as readily found. Fuller [6] considers capability to consist of various elements. The “upper limit” of capability, or competence, is a result of multitude of biological and acquired characteristics, such as motor coordination, information processing capacity and understanding of traffic dynamics through driving experience and training. The actual capability in any given situation is affected also by more transient aspects such as fatigue, motivation and distraction.

In experimental settings driver capability is generally manipulated using a distracting secondary task. Due to obvious road safety implications, the tasks are often naturalistic ones, such as mobile phone handling or usage of in-vehicle navigation systems [2]. But like capability, distraction consists of multiple kinds of phenomena, often divided to visual distraction (“eyes-off-the-road”), cognitive distraction (“mind-off-the-road”) and manual distraction (“hands-off-the-wheel”) [13]. Naturalistic tasks tend to blend some or all of these, which makes them rather difficult to identify and isolate for rigorous analysis. In order to directly operationalize distraction, we focus on the visual distraction using the *occlusion method*, where the driver’s field of view is occluded unless they make a glance by briefly removing the occlusion. This paradigm has a long history in driver behavior research, although it has been used almost exclusively in investigating steering tasks with emphasis on lateral control [14–17].

In sum, to directly measure time headway (task demand) and visual sampling (capability), we developed a driving simulator setting for a car following task, with “eyes-off-the-road” distraction simulated using the occlusion method. The data is used to experimentally examine the assumptions of Hoogendoorn et al. [11] and Saifuzzaman et al. [10]. The results are also discussed in terms of how quantitative behavioral data from simulated and real driving can be incorporated into the current car following models in engineering—and to provide a more solid footing for theoretical ideas in traffic psychology.

## Methods

### Participants

A convenience sample of 18 subjects (9 M, 9 F, age 21 y–35 y, mean 26 y) participated in the study. The participants had held a driving license for an average of 7.6 years (SD 4.1 years), including 2 participants with no driving license. Participants were recruited through personal contacts and university mailing lists.

An informed consent to participate was obtained electronically from each participant as part of the questionnaire. This was done, in accordance with the instructions of the ethics committee, in the form of a fixed-format consent form explaining the purpose of the study, the procedure, and intended use of the data (for scientific purposes only). The study was conducted following the research ethical guidelines of Finnish National Advisory Board on Research Ethics and Helsinki Ethical Review Board in the Humanities and Social and Behavioural Sciences. As per the guidelines, ethical review for the experiment was waived, as the experiment didn’t include any of the criteria that warrant for ethical review. Apart from e-mail address, no identifying information was gathered in the study. The e-mail addresses were removed from the data set by JP in the first step of preprocessing and were stored only on the data logging computer and JP’s workstation.

### Driving simulator

**Fig 1:**
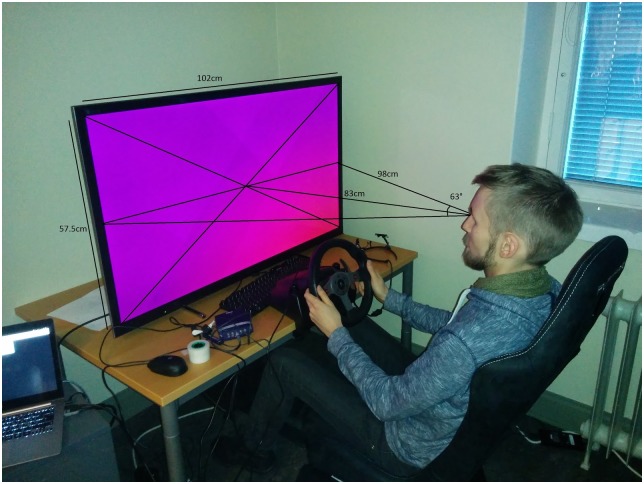
The physical setup of the driving simulator.

The driving simulator software was developed in-house and is available under an open source license [18]. The experiment was run in a fixed based simulator set-up, comprising of a 46 inch display (Sony KDL-46EX653), a distance-adjustable gaming chair (Playseat Evolution Alcantara, Playseats B.V., The Netherlands) and a steering wheel game controller (Logitech G25, Logitech, Fremont, CA). The steering wheel and the gaming chair were in line with the horizontal mid point of the screen, and the vertical mid-point (virtual horizon) was approximately at the eye height (the chair had no height adjustment, so the exact eye height varied with the size of the participant). The viewing distance was about 75–85 cm (depending on the distance adjustment preference of the participant and how upright they would sit), giving the participants a vertical field of about 60–70 degrees (see Fig 1). The simulation was rendered at 1920x1080 pixel resolution targeting 60Hz frame rate with a virtual camera projection configured to have a 65 degree vertical field of view.

The simulated vehicle dynamics parameters were decided by informal pilot testing to give a comfortable compromise of good controllability but not overly nervous responses. For detailed parametrization, see [18] tag v1.1, file vehicle.ls. Steering was disabled in all tasks and the participants only controlled the vehicle’s speed with gas and brake pedals.

### Procedure

Online version of the experiment is available at https://jampekka.github.io/attadapt-demo/. Apart from using keyboard controls, the online version is identical with what was used in our laboratory experiments.

Each session started with obtaining an informed consent and background information with a computer-based questionnaire. This was followed by four different tutorial scenarios simulating basic speed control subtasks, which allowed the participant to learn the simulator’s controls, virtual vehicle dynamics and dimensions and controlling the speed. The tutorial scenarios were added as an effort to minimize learning effects in the final trials that were analyzed. After the tutorial scenarios, two successful practice trials of *unoccluded following task* and *occluded following task* were run. If there was a crash or the participant ran the traffic light signaling beginning of the trial, the practice trials were retried until there was two successful runs, or five trials in total. These were followed by four trials of both tasks in randomized order.

**Fig 2:**
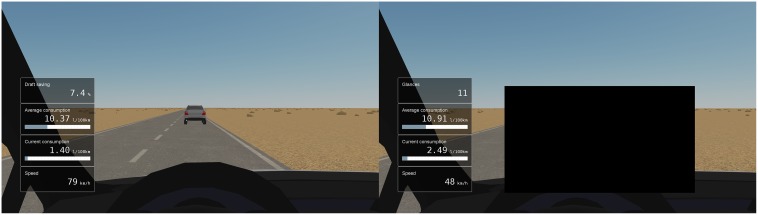
Screenshots of unoccluded car following (left) and occluded car following (right). In the occluded car following scenario the driver could request a visual sample of 300 ms by pressing a paddle in the steering wheel controller.

The instruction for the unoccluded following task (Fig 2 left panel) was to minimize the fuel consumption while driving behind the car ahead. The fuel consumption instruction was chosen to avoid excessive accelerations and decelerations not usually observed in normal driving. However, to promote shorter time gaps, a “draft saving” element was included where the consumption decreased as a function of the distance to the lead vehicle. Meters displaying landspeed and average and instantaneous consumption were shown throughout the tasks. In the normal following task, a draft saving percentage was also shown. For the occluded task this was replaced with number of glances. Draft saving was not shown during the occluded task, as it could be used to deduce the distance to the following vehicle.

In the occluded car following task, a black rectangle (Fig 2, right panel) was placed as an occlusion so that it masked the driver’s own lane and the position of the leading vehicle. The participant could “lift” the occlusion by pressing a lever in the steering wheel, after which the mask was removed for a 300 ms “glance”, after which the occlusion returned. The participants were instructed to minimize fuel consumption as in the normal task, but with minimal number of occlusion removals (glances). No explicit weighting was given for the fuel consumption and the number of glances.

**Fig 3:**
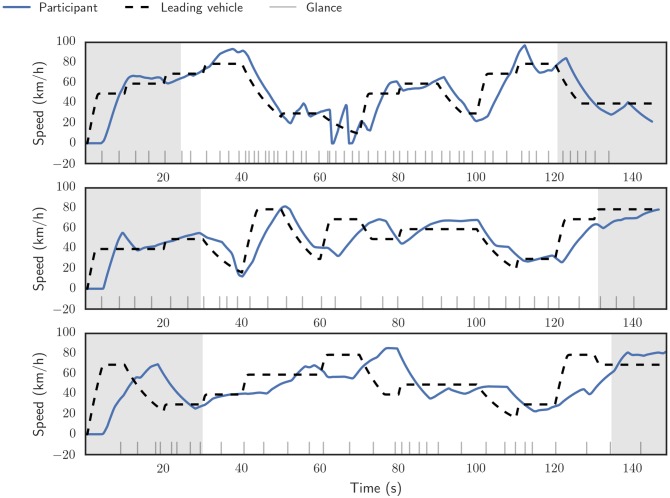
Sample trials of the occluded car following task, showing the speed profile of the the leading vehicle (dashed black line) and the participant’s vehicle (solid blue line). The vertical lines at the bottom represent the glance onset times, points in time when the participant requested a visual sample by removing the occlusion. The shaded areas, corresponding to the first and last 300 meters in distance, were omitted from the analysis. The sample runs are, from top to bottom, 25th, 50th and 75th percentile by average time gap.

The leading vehicle drove with a randomized speed profile, where a target speed was randomly sampled (without replacement) every ten seconds from a set of 0, 30, 40, 50, 60, 70 and 80 km/h. The leading vehicle accelerated or decelerated to this speed using a simple proportional control algorithm (Fig 3). The trial ended when the player had progressed 2000 meters.

### Data preprocessing and analysis

The data analysis was done using custom Python scripts which, along with the data, are available under an open source license [19].

The first 300 meters of driving was omitted from the analysis to discard the initial acceleration from standstill, and the last 300 meters were omitted as some of the participants “strategically” coasted to the end of the trial when they saw the finish line approaching (Fig 3). The somewhat arbitrary criteria of 300 meters on both ends was decided upon visual inspection of the individual trial time series. To prevent very high time gap values, samples where ground speed was under 1.0 m/s were omitted.

### Modeling assumptions

Both Hoogendoorn et al. [11] and Saifuzzaman et al. [12] assume a relationship between driver capability and preferred time headway. We assume that a driver’s *preferred time headway* *T*′(*t*) can be reasonably estimated as a geometric mean of the time headway time series: $T^{\prime }\left(t\right)\approx \hat{T}\left(t\right)$. In the between-subjects analyses we use per-subject differences of average time headway in the occluded tasks and unoccluded tasks: $\Delta \hat{T}\equiv {\hat{T}}_{D}-{\hat{T}}_{0}$, where ${\hat{T}}_{0}$ is occluded average and ${\hat{T}}_{D}$ is unoccluded average.

Both articles leave open how to operationalize the capability *C*(*t*). We assume that capability is inversely related to distraction: $C\left(t\right)={\left(S\left(t\right)+C_{0}^{-1}\right)}^{-1}$, where *C*_0_ is “base capability”, ie capability when distraction *S*(*t*) is zero. We further propose that in our experiment, the geometric mean of occlusion durations approximates driver’s average (visual) distraction: $\hat{S}\left(t\right)\approx \hat{o}\left(t\right)$.

Hoogendoorn et al. [11] formulate task difficulty as *m*_*d*_(*t*) = *m*_*t*_(*t*) − *m*_*c*_(*t*), where *m*_*t*_(*t*) is task demand and *m*_*c*_(*t*) is driver capability. In their model, the preferred time headway increases as a function of task difficulty and “default time headway” $T_{H}^{\prime }$ as: $T^{\prime }\left(t\right)=T_{H}^{\prime }\left(m_{d}{\left(t\right)}^{3}+1\right)$. It is not immediately obvious to us how to quantitatively formulate this in terms of occlusion durations, but it seems to imply that the increase in time headway due to distraction is relative to the headway in the unoccluded task. Thus we assume that Hoogendoorn et al. [11] qualitatively implies a “baseline relative” relationship:

$$\Delta {\hat{T}}_{D}/{\hat{T}}_{0}∼{\hat{o}}_{D}.$$

The formulation of Saifuzzaman et al. [12] doesn’t include an explicit driver capability term, but they assume that “driver capability is inversely proportional to driver’s desired time headway selection”. We interpret this as $\hat{T}\left(t\right)\approx c\hat{C}{\left(t\right)}^{-1}$, where $\hat{C}\left(t\right)$ is average capacity in some neighborhood of *t* and *c* is some constant. This results in a relationship between the physical quantities average occlusion duration and average time headway: $\hat{T}\left(t\right)\approx c\left(\hat{o}\left(t\right)+C_{0}^{-1}\right)$. Assuming that distraction is zero in the normal following task leads further to:

$$\hat{T}\left(t\right)\approx c\hat{o}\left(t\right)+{\hat{T}}_{0}.$$

Thus an increase in time headway is directly proportional to increase in occlusion duration: $\Delta \hat{T}\approx c{\hat{o}}_{D}$. More generally, and in contrast to Hoogendoorn et al. [11], this implies “baseline independent” relationship:

$$\Delta \hat{T}∼{\hat{o}}_{D}.$$

## Results

### Average occlusion duration and time headway

As discussed in the methods, we interpret that Hoogendoorn et al. [11] proposes “baseline relative” relationship, $\Delta \hat{T}/{\hat{T}}_{0}∼\hat{o}$, where increase in time headway due to visual distraction depends on the time headway on non-distracted “baseline” task, whereas Saifuzzaman et al. [12] is interpreted to assume “baseline independent” relationship, where time headway increase is only a function of the visual distraction.

In our data, the “baseline independent” relationship between average time headway and average occlusion duration yields a Spearman correlation of 0.84 (95% CI (0.62, 0.94)) whereas the “baseline relative” yields a lower correlation of 0.57 (95% CI (0.14, 0.82)). The difference has 95% CI of (0.026, 0.66) (using method of Zou [20]), indicating that the “baseline independent” formulation results in a significantly stronger qualitative relationship.

**Fig 4:**
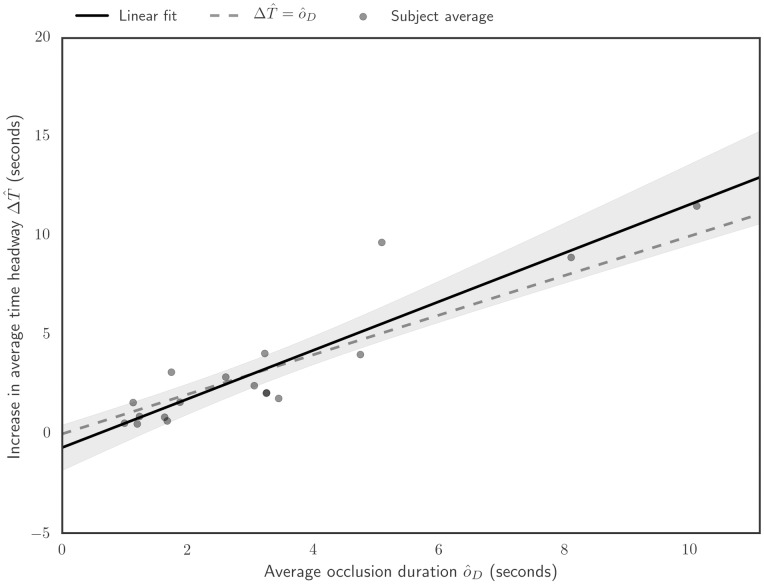
Time headway increase in occluded driving relative to unoccluded car following, as a function of the participant’s average occlusion duration. Each dot indicates an individual participant. When the occlusion task is introduced to the experiment, the participants leave a longer time headway and the individual participants choose idiosyncratic “trade offs” between leaving more headway vs. requesting samples. At a group level, however, the trade off is well described by a linear relation (solid black line). The case of time headway increase being equal to the glance duration (dashed black line) can not be ruled out on 95% confidence level (gray shaded area).

Further studying the relationship $\Delta \hat{T}∼{\hat{o}}_{D}$ shows it to be quite linear, with the geometric average occlusion duration ${\hat{o}}_{D}$ explaining 84% of variance of the geometric average time headway (Fig 4). Furthermore, the “trivial” case of $\Delta \hat{T}={\hat{o}}_{D}$ cannot be ruled out on 95% confidence level, which quantitatively corroborates the formulation $\hat{T}\left(t\right)\approx c\hat{o}\left(t\right)+{\hat{T}}_{0}$, with *c* ≈ 1, ie that average time headway increases in approximately one-to-one wrt. average occlusion duration.

**Fig 5:**
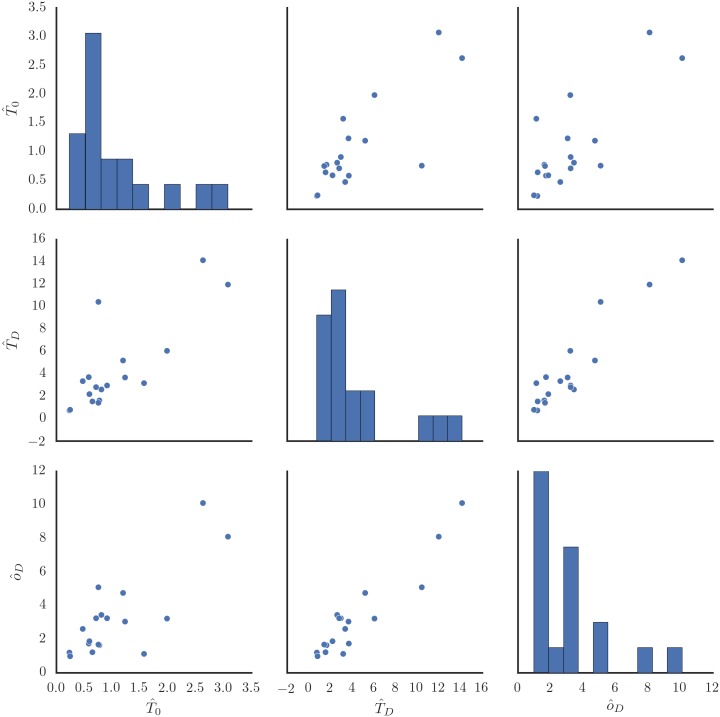
Per-subject geometric means of unoccluded time headway ${\hat{T}}_{0}$, occluded time headway ${\hat{T}}_{D}$ and occlusion duration ${\hat{o}}_{D}$.

However, it should be noted that the choice of the particular form of ${\hat{T}}_{D}-{\hat{T}}_{0}\approx \alpha \hat{o}+\alpha_{0}$ is somewhat arbitrary, and selected largely due to simplicity and compatibility with previous modeling efforts. All of the variables in the equation correlate rather strongly with each other (Fig 5), making model selection underdetermined by data alone.

With these caveats, based on our results, we propose that $\hat{T}\left(t\right)\approx {\hat{T}}_{0}+\hat{o}\left(t\right)$ can be used as a reasonable approximation for preferred time gap under “eyes-off-the-road” type visual distraction. The estimate for unoccluded preferred time gap ${\hat{T}}_{0}\approx T^{\prime }$ has quite substantial between-subject variation (Fig 5), but as a first approximation one second could be used.

### Instantaneous Time Headway and Occlusion Duration

**Fig 6:**
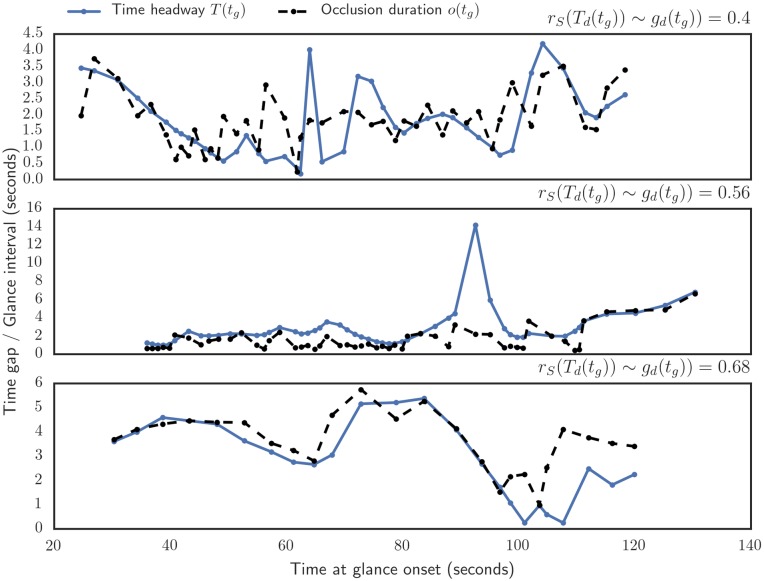
Sample time series showing time headway and next occlusion duration at glance onset moments. The sample runs are, from top to bottom, 25th, 50th and 75th percentile by Spearman correlation between the detrended instantaneous time gaps and occlusion durations.

To further study the relationship between time headway and occlusion duration at within-subject and within-trial level, we sample both signals only at “glance onset moments” *t*_*g*_, ie time instances where the subject lifts the blinder. At such sampled signal, occlusion duration *o*(*t*_*g*_) measure how long the subject chooses to drive without visual input after the glance and “instantaneous time headway” *T*(*t*_*g*_) measure time gaps at the glance onset moments (Fig 6).

To reduce possible spurious relationship due to within-subject and within-trial variation in the preferred time gap *T*′(*t*) and preferred glance interval *o*′(*t*), we subtract a robust linear trends $\tilde{T}\left(t_{g}\right)$ and $\tilde{o}\left(t_{g}\right)$ from both signals, resulting in $T_{d}\left(t_{g}\right)=T\left(t_{g}\right)-\tilde{T}\left(t_{g}\right)$ and $o_{d}\left(t_{g}\right)=o\left(t_{g}\right)-\tilde{o}\left(t_{g}\right)$

We find a robust qualitative relationship between time headway and occlusion duration also for these instantaneous values. From a total of 61 trials, 58 had a positive *T*_*d*_(*t*_*g*_) ∼ *o*_*d*_(*t*_*g*_) Spearman correlation (Binomial test *p* = 3.3 × 10^−14^). The relationship was also consistent between subjects: for all 18 subjects the median correlation of the trials was positive (Binomial test *p* = 7.6 × 10^−6^). The relationship is also rather strong: the overall median Spearman correlation was 0.56 and median of subject medians was 0.57.

As neither of the variables are under experimental control and the order of causation is unclear, we use an “error-in-variables” type symmetric regression for parameter estimates to avoid bias due to regression attenuation. Furthermore, as the error variances in the variables aren’t known, we opt for the non-parametric and symmetric Passing-Bablok regression [21].

**Fig 7:**
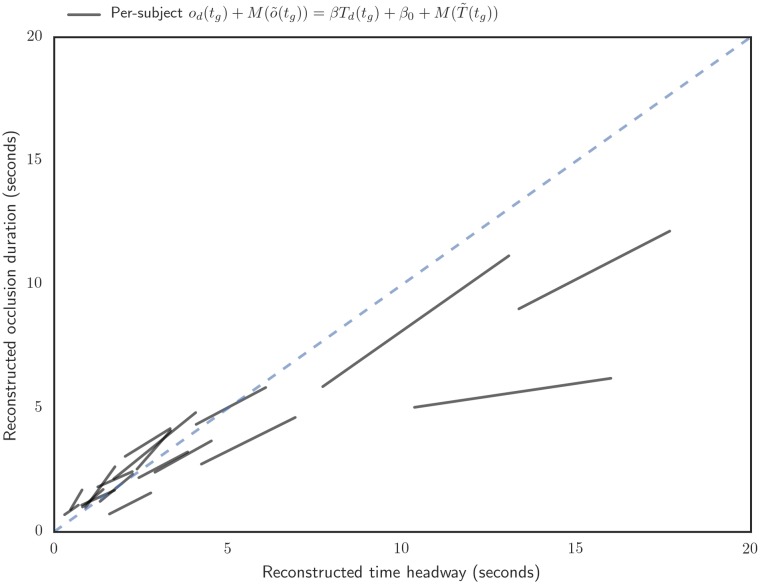
Per-subject Passing-Bablok regression estimates between detrended instantaneous time headways and occlusion durations, with median of each subject’s trend added back to predicted values. Regression line x-axis range for each subject is from 25th to 75th percentile.

Per-subject Passing-Bablok regressions show some variation in the slope, with the median at 0.83 (Fig 7). Intercepts are naturally quite close to zero (median -0.05) due to the detrending.

Based on visual inspection of the time series (Fig 6), and the correlation surviving the detrending, we posit that this correspondence between the time headways and occlusion durations occurs on a rather short timescale, on the order of seconds. We propose that on this short time scale the covariation is mostly due to the driver momentarily adapting their task capability to the situation’s demands, ie when the time gap is large/small, the driver can have their “eyes off the road” for a longer/shorter duration.

Assuming this direction of causality, the result suggests that *g*_*d*_(*t*) ≈ *βT*_*d*_(*t*), with *β* typically around 1.0, could be used as a crude approximation of how an average driver adapts visual sampling to transient changes in time headway. Assuming that the linear trends of time gap and inter glance interval approximates the between subjects and longer timescale preferences time headway $T^{\prime }\left(t\right)\approx \tilde{T}\left(t\right)$ and occlusion durations $o^{\prime }\left(t\right)\approx \tilde{o}\left(t\right)$, this can be further expanded:

$$\begin{array}{cc} o\left(t\right)-o^{\prime }\left(t\right) & \approx \beta (T\left(t\right)-T^{\prime }\left(t\right))\\ o(t) & \approx \beta \left(T\left(t\right)-T^{\prime }\left(t\right)\right)+o^{\prime }\left(t\right). \end{array}$$

## Discussion

In summary, our data shows a robust and strong relationship between the time headway to the leading vehicle and the accepted occlusion duration. This dependency is present across different levels of time-scale, and at between-subjects and within-subject levels.

Our interpretation is that the different time scales may reflect slightly different processes, at different levels of hierarchical analysis of the driving task [22]. The between-subjects dependency at longer time scale may reflect a “strategic” or “tactical” individual preference in setting their desired headway or glance interval, i.e. the trade-off between achieving the instructed intermittency of visual updating while maintaining sufficient control of safety margins. The within-subject dependency at the shorter time-scale may in turn may reflect “operational” level feedback responses to unpredictable changes in the leading vehicle speed. If the leading vehicle slows down (especially during occlusion) and thereby ends up closer than desired, the driver increases visual sampling frequency as they slow down. If, on the other hand, the leading vehicle accelerates rapidly the time headway will increase, and there is less need for visual sampling and the occlusion duration will increase.

In addition to showing the existence of the relationships, we provided tentative approximations of their quantitative forms. The approximations, although subject to various limitations, can hopefully be utilized in traffic safety policy discussion, quantitative modeling of driver behavior, and importantly as null hypotheses to be challenged and refined in future work.

### Limitations

#### Ecological validity of the simulator

Simulator based results are naturally subject to the many general problems of how well a simulator replicates real-world driving tasks [23]. Some discrepancies are already apparent: the typical time gaps in the normal following task were somewhat smaller than are found in real-world measurements. There are some deliberately made choices in the task that are expected to yield smaller time gaps: the subjects were encouraged to drive with small time gaps, the task didn’t include steering, the lead vehicle’s decelerations were quite subtle and the structure of the road environment was extremely simple. But there is also quite a clear reason to believe that driving was significantly more “risky” than it would be with a real car: from the total of 144 trials 9 ended with a crash, which would translate to about 31000 crashes per 100 million kilometers driven, which is of course several magnitudes higher than real world traffic accident rates; the 2014 finnish rate of accidents ending in insurance claims was about 170 accidents per 100 million kilometers driven [24].

Also the instruction used, minimizing fuel consumption, is somewhat unconventional. We opted for this instruction based on pilot testing, where instruction to drive with minimal (time) headway often led to a “racing game like” driving, with unrealistically high accelerations, and the common instruction “drive as you normally would” led to very long headways, which would be problematic with our modeling assumption that the participants drove with very low distraction levels ($C_{0}^{-1}$) in the normal following task. The “fuel consumption” framing of the task instruction was deemed a satisfactory way to instigate optimizing behavior without encouraging aggressive driving. However, the instruction does cause an extra component for the participants to monitor and control, which may somewhat change the dynamics of the task in comparison to normal real-world driving.

Examining ecological validity of the results with real-world driving experiment is important future work. Fortunately this should be relatively straightforward as the methological foundations are already laid in previous visual occlusion studies.

#### Occlusion duration as index of distraction

The occlusion paradigm has favorable methodological properties, but of course the dynamics of distraction and capability in the real world are much more complex. Attention during driving isn’t only about taking visual input in discrete time intervals. Many, or even most, types of distraction, such as drowsiness or secondary tasks have significant or even dominating *cognitive distraction* component, ie the eyes may well be in the road ahead, yet the information is not sufficiently processed or reacted to.

#### Non-representative sample of participants

Our convenience sample of participants clearly are not a representative sample of drivers: they were significantly younger than the general driving population and all didn’t even have a driving license. This naturally brings about concerns about the generalizability of the quantitative results, but the robustness of the results is quite a strong indication that they should generalize to the general population at least on a qualitative level. We also conducted time headway analyses using difference from the unoccluded task ($\Delta \hat{T}$), which we assume will generalize better than absolute time headway values.

#### Simplistic parameterization of task demand

In our analyses, task demand is operationalized only as function of time headway. Quite obviously, this doesn’t capture the entirety of a driving task’s demands, even in the extremely reduced car following task used in our experiment. The rather strong results in between-subject analyses indicate that when averaged over long spans of driving, time headway seems to index task demand quite well, if measured by frequency of glances.

The instantaneous case, while statistically quite robust, leaves plenty of variation to be explained. Also we use non-parametric methods for analyses of the instantaneous case, which leaves open the distributional characteristics of the unexplained variation. More accurate account of how the task demand varies as a function of the driving situation is clearly needed for realistic modeling of the demand-capability dynamics.

## Conclusions

On qualitative level the results support both Hoogendoorn et al. [11] and Saifuzzaman et al. [12] in that average time headway significantly changes in response to distraction, but the latter better explains our observations. Furthermore their proposal “driver capability is inversely proportional to driver’s desired time headway selection” interpreted as $\hat{T}\left(t\right)\approx c\hat{C}{\left(t\right)}^{-1}$ explains our data remarkably well when the average capability is operationalized using the average occlusion duration and unoccluded driving performance: $\hat{C}\left(t\right)\approx {\left(\hat{o}\left(t\right)+{\hat{T}}_{0}\right)}^{-1}$.

However, we also find that there’s similar relationship in the shorter, non-averaged, timescale. We propose that this is largely due to drivers adapting their momentary visual attention to fluctuations in task demand due to momentary deviations from the “desired time gap”. Such adaptive attention is observed in everyday experience and in large scale field studies [3, 25]. This kind of adaptation isn’t incorporated in the discussed TCI-CF models, and more generally majority of existing car following models effectively assume constant, or slowly changing, monitoring and vigilance.

The traffic simulator HUTSim does include a CF model where the update rate of the model is reduced during periods of uneventful driving, which can lead to collisions or near-collisions [26]. Yang et al. [27] developed a car following model in which the modeled driver reaction time fluctuates as a result of distraction, with intermitted sampling, or “eyes-off-the-road” distraction as one component. Przybyla et al. [28] developed methodology to indirectly estimate driver distraction using reaction times from naturalistic data. Our data and results provide experimental insight for evaluating such models, and bridging them with the TCI and adaptive time headway models based on it.

The occlusion paradigm may prove to be useful setting for providing more rigorous operationalizations for traffic psychological theories. In fact our assumptions do imply such operationalizations for the TCI: at least in a simplified car following task, capability and task demand are operationalized as inverse of seconds, with occlusion duration and time headway providing measurable estimates. The close connection between the occlusion duration and time headway can also provide a tool to operationalize more complex distractions, which could be estimated by their effect on the driving performance.

However, the TCI, while elegant yet explaining variety of phenomena, is rather coarse view of driver behavior. Deeper understanding of the driving process and more general models will undoubtedly require refining what high level constructs like task demand and capability “are made of” [29]. Although the TCI forwarded common ground between previously seemingly conflicting ideas, the theoretical and experimental work of understanding the mechanisms underneath is far from finished [23].

In the experimental field, the occlusion paradigm, especially in the short time scale, could also provide a bridge between research in speed control and steering, which are currently somewhat separate fields of inquiry. The connection to previous occlusion studies, concerning mostly lateral control (steering) [14–17], is readily apparent. This connection might prove fruitful in bringing the models of visual control of an automobile [30–33] (for review see [34]) closer to traffic engineering car following models. It would therefore be desirable to extend the current paradigm and modeling efforts to more complex scenarios involving both lateral and longitudinal control on curved roads with more visual 3D structure. Concretely combining insights in various fields of traffic psychology and traffic engineering would surely help in moving towards a quantitative and general model of driver behavior.
